# Engineering *Cercospora* disease resistance via expression of *Cercospora nicotianae* cercosporin-resistance genes and silencing of cercosporin production in tobacco

**DOI:** 10.1371/journal.pone.0230362

**Published:** 2020-03-16

**Authors:** Elizabeth Thomas, Sonia Herrero, Hayde Eng, Nafisa Gomaa, Jeff Gillikin, Roslyn Noar, Aydin Beseli, Margaret E. Daub

**Affiliations:** 1 Department of Plant and Microbial Biology, North Carolina State University, Raleigh, NC, United States of America; 2 Botany Department, Faculty of Science, Fayoum University, Al Fayoum, Egypt; University of Nebraska-Lincoln, UNITED STATES

## Abstract

Fungi in the genus *Cercospora* cause crop losses world-wide on many crop species. The wide host range and success of these pathogens has been attributed to the production of a photoactivated toxin, cercosporin. We engineered tobacco for resistance to *Cercospora nicotianae* utilizing two strategies: 1) transformation with cercosporin autoresistance genes isolated from the fungus, and 2) transformation with constructs to silence the production of cercosporin during disease development. Three *C*. *nicotianae* cercosporin autoresistance genes were tested: *ATR1* and *CFP*, encoding an ABC and an MFS transporter, respectively, and *71cR*, which encodes a hypothetical protein. Resistance to the pathogen was identified in transgenic lines expressing *ATR1* and *71cR*, but not in lines transformed with *CFP*. Silencing of the *CTB1* polyketide synthase and to a lesser extent the *CTB8* pathway regulator in the cercosporin biosynthetic pathway also led to the recovery of resistant lines. All lines tested expressed the transgenes, and a direct correlation between the level of transgene expression and disease resistance was not identified in any line. Resistance was also not correlated with the degree of silencing in the *CTB1* and *CTB8* silenced lines. We conclude that expression of fungal cercosporin autoresistance genes as well as silencing of the cercosporin pathway are both effective strategies for engineering resistance to *Cercospora* diseases where cercosporin plays a critical role.

## Introduction

Fungi in the genus *Cercospora* cause devastating crop losses on a wide range of crop plants world-wide including sugar beet, corn, and coffee as well as many vegetable and ornamental species [1]. The wide host range and lack of disease resistance in many host species has been attributed to the fungus’ production of a photoactivated perylenequinone toxin, cercosporin (for review see [2–4]). Cercosporin is a photosensitizing compound that absorbs light energy to generate singlet oxygen (^1^O_2_) as well as other reactive oxygen species (ROS) [5–8]. In host plants the cercosporin-generated ROS cause peroxidation of the host cell membrane lipids, leading to membrane breakdown, death of host cells and leakage of nutrients needed by the fungus for tissue colonization [9–11]. Studies in several *Cercospora*-host systems have documented the importance of cercosporin in disease by documenting reductions in disease development by mutants deficient for cercosporin production [12–17]. The importance of light, required for cercosporin photoactivation, in disease symptom development has also been documented in both coffee and sugar beet [18, 19].

Singlet oxygen is a non-radical, but highly reactive species of ROS [4]. Unlike free-radical forms of ROS such as superoxide and hydrogen peroxide, singlet oxygen is not a common part of cellular reactions, and most cells lack effective resistance mechanisms. Compounds have been identified that quench singlet oxygen [20]. Carotenoids are among the most effective quenchers of singlet oxygen, and play a role in defense against singlet oxygen generated as part of photosynthesis [21]. But there is little evidence for a role for quenchers in resistance to photosensitizer toxicity in cells [3]. Consistent with its production of singlet oxygen, cercosporin has been shown to be almost universally toxic, with toxicity documented not only to plants, but also to mice, bacteria, oomycetes, and many fungi [8, 22].

Although cercosporin has been shown to be almost universally toxic, *Cercospora* species are resistant to its toxicity [22]. Thus, extensive studies have been conducted to characterize autoresistance mechanisms in *Cercospora* fungi as a strategy to identify genes that may have utility in engineering cercosporin-resistant plants. Characterization of endogenous quenchers including carotenoids, as well as antioxidant enzymes and general antioxidant activity were not correlated with cercosporin resistance in fungi (for review see [3, 23]). Vitamin B6 production was correlated with autoresistance, however expression of *Cercospora* B6 biosynthetic genes (*PDX1*, *PDX2*) in tobacco did not elevate B6 levels or cercosporin resistance due to down-regulation of the endogenous tobacco *PDX* genes [24]. Identification of the zinc cluster transcription factor CRG1 that regulates cercosporin resistance and production [25] led to the recovery of putative resistance genes through the use of a subtractive hybridization strategy of genes differentially expressed between the wild type strain and a *crg1* mutant [26]. Differentially regulated genes identified through this strategy have subsequently been characterized for a possible role in resistance through characterization of mutants [27] as well as by the ability to impart cercosporin resistance to the cercosporin-sensitive fungus *Neurospora crassa* [28, 29].

Another strategy for engineering resistance is to use host-induced gene silencing (HIGS) with RNA interference (RNAi) to silence expression of critical pathogenicity genes. The utility of HIGS to protect plants against fungal pathogens was first demonstrated by Nowara et al. [30] with the obligate powdery mildew fungus *Blumeria graminis*. It has since been shown to function against fungi with diverse modes of pathogenicity, including other obligate pathogens such as rusts [31], as well as species of *Fusarium* [32], *Verticillium* [33], and *Sclerotinia* [34] (for review see [35]). The biosynthetic pathway for cercosporin production has been characterized. The polyketide synthase (PKS) in the pathway was first identified through recovery from cercosporin-deficient mutants of *C*. *nicotianae* created by restriction enzyme-mediated insertion (REMI) [13]. Other genes in the pathway were then identified through sequencing of flanking regions. This work identified a cluster of eight genes (*CTB1*-*CTB8*) encoding methyltransferases, oxidoreductases, a transporter, and a transcription factor in addition to the PKS [36]. In addition, a recent genomic study of *C*. *beticola* found five additional loci flanking the cluster (*CTB9-12*) that have a role in biosynthesis [37]. Characterization of cluster genes has led to the identification of the metabolic intermediates involved in cercosporin production [38].

Here we report on the transformation of tobacco to express three autoresistance genes (*ATR1*, *CFP*, and *71cR*) identified through our subtractive hybridization work with the *crg1* mutant [26], as well as silencing constructs targeting the *CTB1* and *CTB8* genes in the biosynthetic pathway [36]. *ATR1* encodes an ABC transporter involved in both cercosporin production and autoresistance [27]. *CFP*, also identified through our subtractive hybridization study, encodes an MFS transporter that was previously identified as a light-induced gene involved in both cercosporin production and resistance [12]. Significantly, the *C*. *beticola* genomic study cited above identified *CFP* as among the flanking loci to the CTB cluster that have a role in cercosporin biosynthesis [37]. Transformation of *CFP* into ‘Xanthi’ tobacco has been previously reported to reduce lesion size caused by *C*. *nicotianae* [39]. The gene *71cR* encodes a hypothetical protein that has been shown to impart cercosporin resistance when expressed in *N*. *crassa* [29]. Efforts to silence the pathway utilized *CTB1*, encoding the polyketide synthase, the first step in the biosynthetic pathway [13], as well as *CTB8*, the pathway regulator [36].

## Results

### Transformation and recovery of transformed lines

*Nicotiana tabacum* cv ‘Hicks’ was transformed via *Agrobacterium*-mediated transformation as previously described [24]. Independent transformations were done with three genes from *C*. *nicotianae* previously shown to be involved in cercosporin autoresistance in the fungus: *ATR1*, encoding a putative ABC transporter [27]; *CFP*, encoding a putative MFS transporter [12, 27]; and *71cR*, encoding a hypothetical protein [29]. Transformants with *ATR1* and *CFP* utilized haploid plants generated by crossing *N*. *tabacum* with *Nicotiana africana* [40]. Bialaphos-resistant transformed plants were regenerated and rooted in culture, and rooted plants were transferred to soil. Plants were screened by PCR to confirm transformation, and then screened for *ATR1* or *CFP* expression using qPCR (S1 Fig). A sub-set of plants showing high levels of transgene expression were selected, and homozygous doubled haploid lines were generated using a mid-vein culture technique as previously described [24]. Plants regenerated from mid-vein culture were grown to maturity in the greenhouse, and doubled haploid plants were selected based on production of pollen and viable seed. In total, 424 haploid *ATR1*-transformed plants were isolated, and 21 lines were selected for chromosome doubling based on gene expression. One fertile doubled haploid plant recovered from each of these 21 haploid transformants was used for further analyses. A total of 244 *CFP* transformed haploid plants were isolated, and 11 were selected for chromosome doubling based on gene expression. One fertile doubled haploid plant from each of the 11 lines was used for further analyses.

*Agrobacterium*-mediated transformation with *71cR* was done on diploid plants of cv ‘Hicks’. In this case, rooted plantlets (T_0_) were grown in soil, and screened by PCR to confirm transformation, and then by qPCR to quantify *71cR* expression (S1 Fig). Five transformed plants, four with the highest levels of expression (Lines 4, 7, 14, and 20) and one with the lowest gene expression (Line 9), were selfed, and T_1_ seed recovered. Seed from each plant was screened *in vitro* for resistance to bialaphos, and 10 resistant seedlings (T_1_) from each of the five T_0_ plants were grown in the greenhouse, allowed to self, and T_2_ seed harvested. The T_2_ seed from the 10 plants of each of the five lines were screened *in vitro* for resistance to bialaphos, and plants producing seed populations that were not segregating for bialaphos resistance (S2 Fig) were scored as homozygous and used for further analyses.

Transformation for silencing of cercosporin production utilized RNAi constructs of two genes in the cercosporin biosynthesis pathway [36]: *CTB1*, encoding the polyketide synthase, the first enzymatic step in the pathway, and *CTB8*, encoding the pathway transcriptional activator. Transformation was done with haploid cv ‘Hicks’ tobacco plants, and plants were selected using a kanamycin-resistance marker. Plants were assayed by semi-quantitative RT-PCR for expression of the transgene construct, and plants with the highest levels of transgene expression were selected for further analyses. Homozygous doubled haploid plants were generated via mid-vein culture as was described above for *ATR1* and *CFP*. In total, 204 haploid plants transformed with the *CTB1* silencing construct were isolated, of which 15 were selected for chromosome doubling based on gene expression; one fertile doubled haploid plant recovered from each of the 15 *CTB1*-transformants was used for further studies. For *CTB8*, 340 transformed haploid plants were isolated, with 15 selected for chromosome doubling. One fertile plant from each of these 15 *CTB8* transformants was selected for further analysis.

### Disease resistance, transgene expression and cercosporin resistance of lines transformed with *ATR1*, *CFP*, and *71cR*

**Fig 1:**
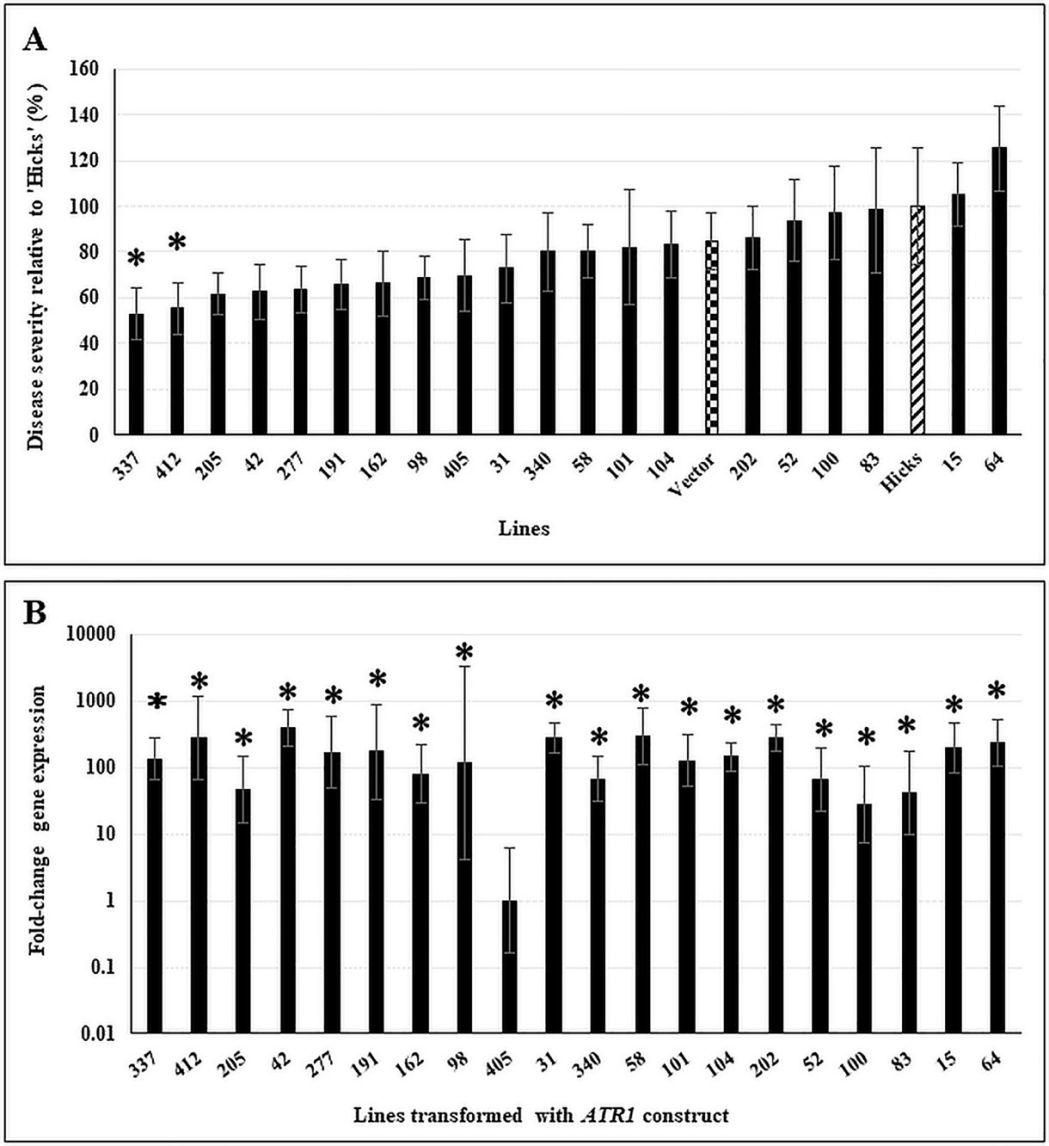
Disease response and transgene expression in *ATR1*-transformed lines. **A.** Disease response of 12-week old *ATR1*-transformed doubled haploid lines inoculated with *C*. *nicotianae*. Controls included a vector-transformed line (checkered bar) and the susceptible tobacco cv ‘Hicks’ (hatched bar). Disease severity was assayed as the number of coalesced lesions at 6 weeks post-inoculation and is shown relative to severity of cv ‘Hicks’. Data shown are results of two independent experiments with 5 plants/line in each experiment. The within-group differences between the different *ATR1*-transformed lines were examined using Satterthwaite-Smith-Welch test. Lines with *p* ≤ 0.05 were considered to be significant (denoted by asterisks). **B.** *ATR1* transgene expression in 10-week old non-inoculated plants. Samples were normalized to tobacco polyubiquitin gene expression. Statistical significance was assessed using one-way ANOVA combined with Dunnett’s multiple comparison analysis using the lowest normalized transcript (Line 405) as the control group. Gene expression was considered statistically significant with *p* ≤ 0.05 (denoted by asterisks). Fold-change gene expression is shown on a log_10_ scale relative to the lowest expressor (Line 405). Error bars indicate standard error from three biological replicates. Each biological replicate was tested with three technical replicates.

**Fig 2:**
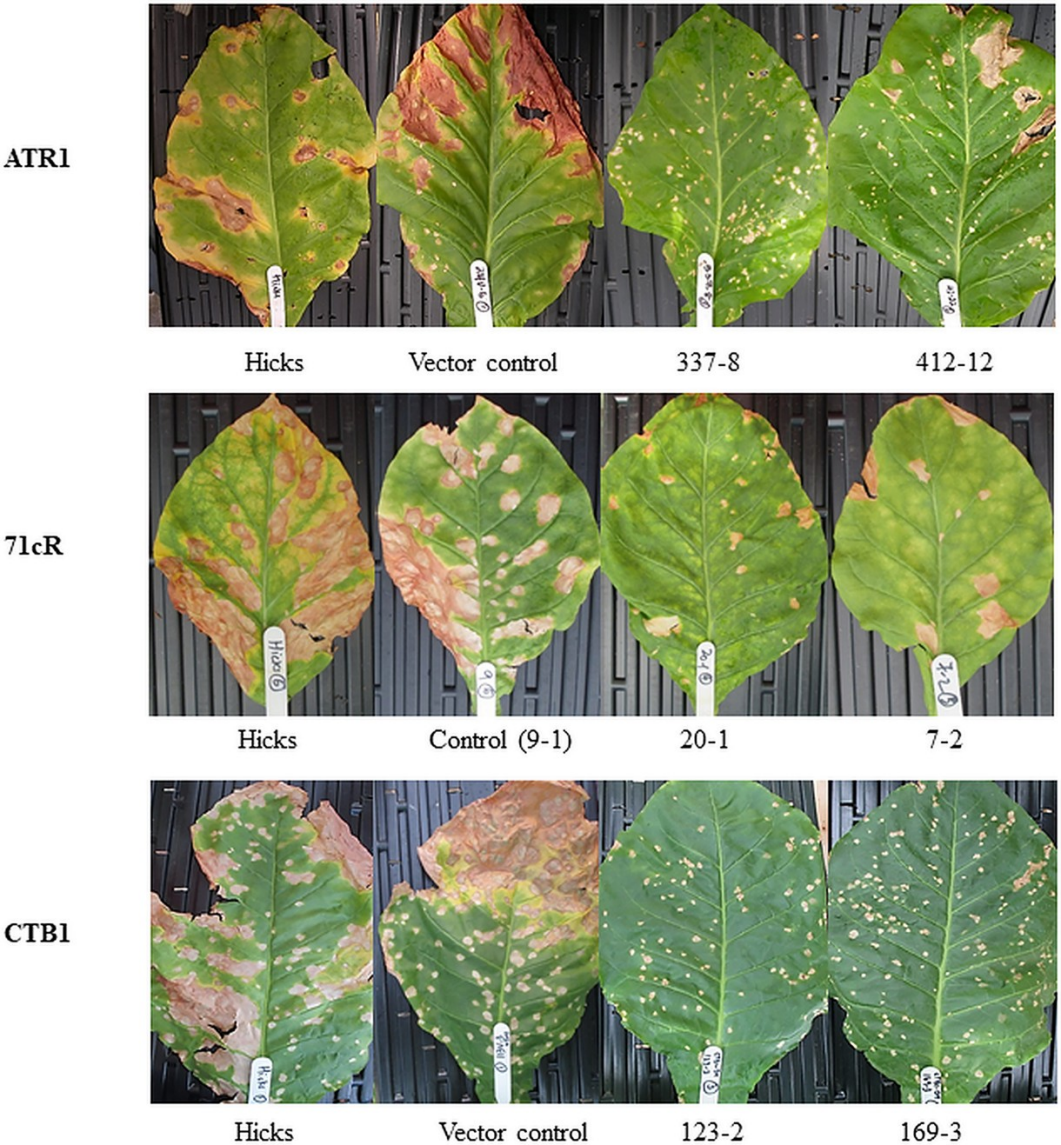
Symptoms on wild-type cv ‘Hicks’ and transformed plants inoculated with *C*. *nicotianae*. **Top:** Resistant *ATR1*-transformed Lines 337 and 412 as compared to symptoms on cv ‘Hicks’ and the vector control. **Middle:** Resistant *71cR*-transformed Lines 20–1 and 7–2 as compared to symptoms on cv ‘Hicks’ and the 9–1 lowest-expression control. **Bottom:** Symptoms on lines 123 and 169 transformed with silencing constructs for *CTB1* as compared to cv ‘Hicks’ and the vector control.

Lines transformed for *ATR1*, *CFP*, and *71cR* were assayed for transgene expression and disease resistance under greenhouse conditions. With *ATR1*, two lines were identified that showed significant reduction in disease symptoms as compared to the control cv ‘Hicks’ (Fig 1A). Infected leaves developed lesions, but the lesions remained small, and did not expand (Fig 2). Differences in symptom development between the transgenic lines and wild type were analyzed using the Sattherwaite-Smith-Welch test [41, 42]. Gene expression analysis (Fig 1B) showed high levels of expression of *ATR1* in all transgenic plants except for line 405. Results of the one-way ANOVA combined with post hoc comparisons of normalized transcripts using Dunnett’s test with Line 405 as the control group, indicated that the expression of *ATR1* construct was statistically significant in all transgenic lines (*p* ≤ 0.05).

**Fig 3:**
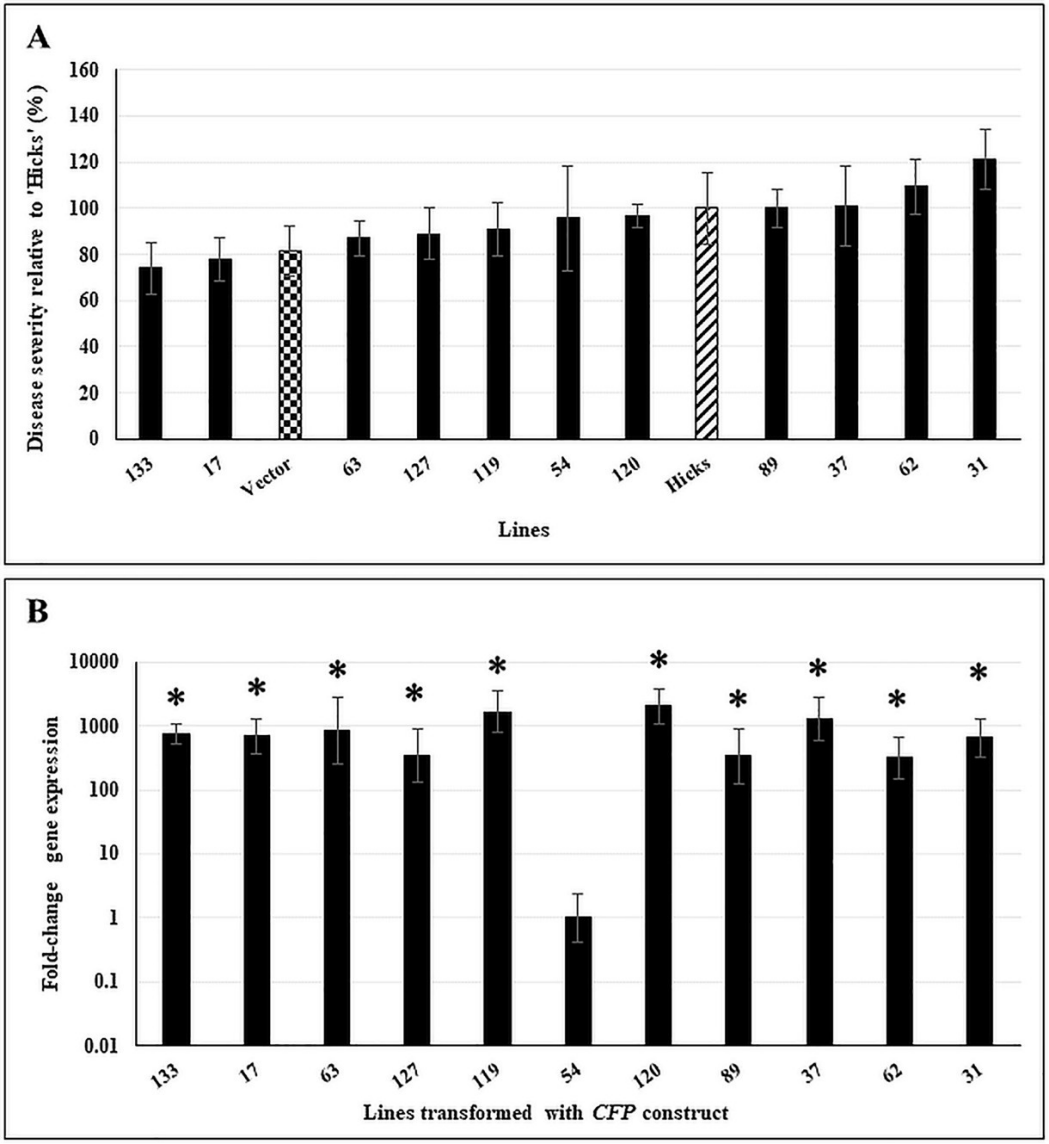
Disease response and transgene expression in *CFP*-transformed lines. **A.** Disease response of 12-week old double haploid *CFP*-transformed lines inoculated with *C*. *nicotianae*. Controls included a vector-transformed line (checkered bar) and the susceptible tobacco cv ‘Hicks’ (hatched bar). Disease severity was assayed as the number of coalesced lesions at 6 weeks post-inoculation and shown relative to severity of cv ‘Hicks’. Data shown are results of three independent experiments with 5 plants/line in each experiment. The within-group differences between the different *CFP*-transformed lines were examined using Satterthwaite-Smith-Welch test. There were no significant differences between lines (*p* ≤ 0.05). **B.** *CFP* transgene expression in 10-week old non-inoculated plants. Samples were normalized to tobacco polyubiquitin gene expression. Statistical significance was assessed using one-way ANOVA combined with Dunnett’s multiple comparison analysis using the lowest normalized transcript (Line 54) as the control group. Gene expression was considered statistically significant with *p* ≤ 0.05 (denoted by asterisks). Fold-change gene expression is shown on a log_10_ scale relative to the lowest expressor (Line 54). Error bars indicate standard error from three biological replicates. Each biological replicate was tested with three technical replicates.

In contrast to *ATR1*, no statistically significant disease resistance was seen in *CFP*-transformed plants relative to controls (Fig 3A). As with *ATR1*-transformed plants, high transgene expression was found in all lines, and was statistically significant as compared to Line 54, used as the control (Fig 3B).

**Fig 4:**
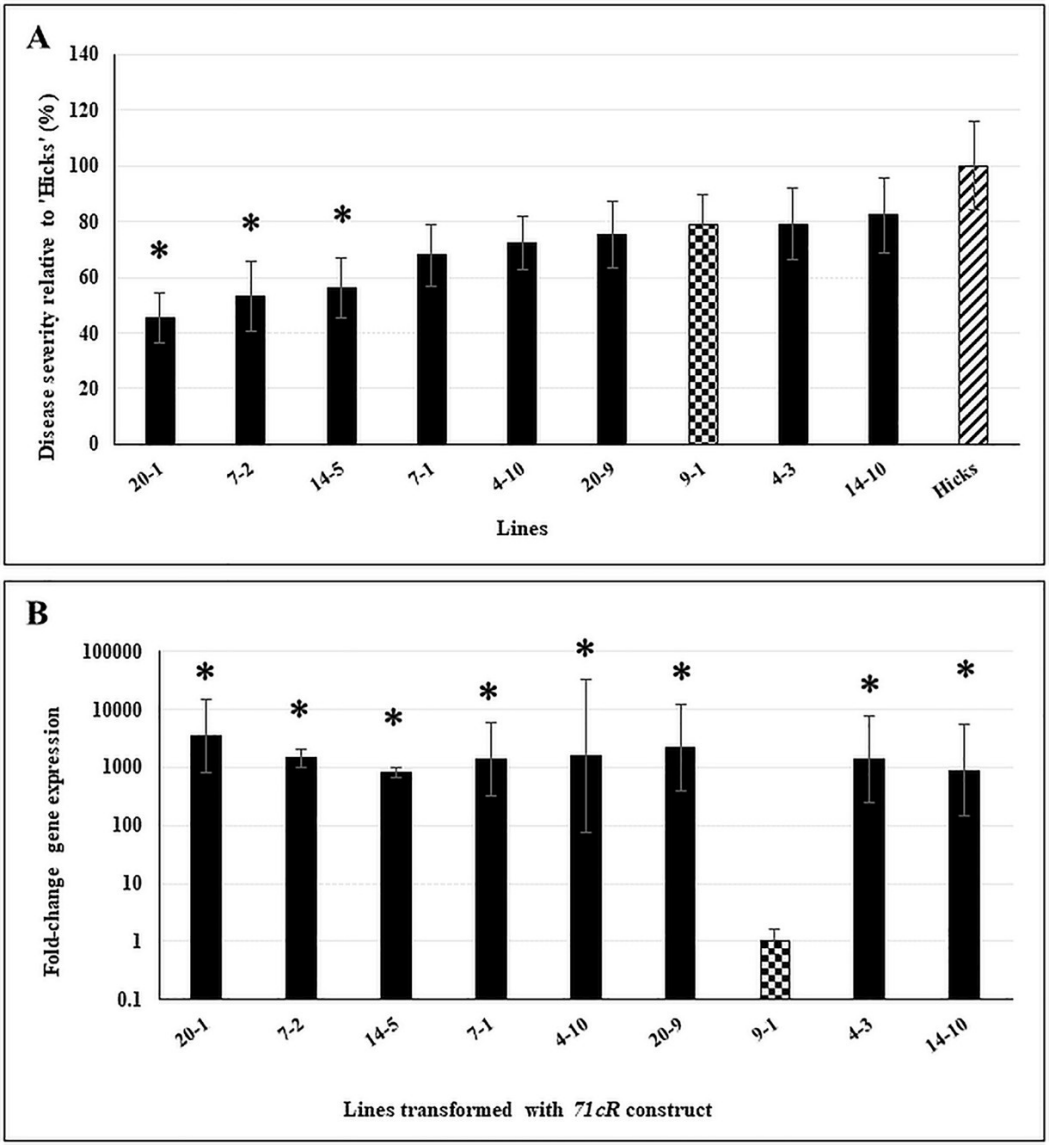
Disease response and transgene expression in *71cR*-transformed lines. **A.** Disease response of 12-week old *71cR*-transformed lines inoculated with *C*. *nicotianae*. Controls included the lowest expressor line 9–1 (checkered bar) and the susceptible tobacco cv ‘Hicks’ (hatched bar). Disease severity was assayed as the number of coalesced lesions at 4 weeks post-inoculation and shown relative to severity of cv ‘Hicks’. Data shown are results of two independent experiments with 5 plants/line in each experiment. The within-group differences between the different *71cR*-transformed lines were examined using Satterthwaite-Smith-Welch test. Lines with *p* ≤ 0.05 were considered to be significant (denoted by asterisks). **B.** *71cR* transgene expression in 10-week old non-inoculated plants. Samples were normalized to tobacco elongation factor 1-alpha gene expression. Statistical significance was assessed using one-way ANOVA combined with Dunnett’s multiple comparison analysis using the lowest normalized transcript (Line 9–1) as the control group. Gene expression was considered statistically significant with *p* ≤ 0.05 (denoted by asterisks). Fold-change gene expression is shown on a log_10_ scale relative to the lowest expressor (Line 9–1, checkered bar). Error bars indicate standard error from three biological replicates. Each biological replicate was tested with three technical replicates.

With *71cR*, three lines were identified that showed significant reduction in disease symptoms as compared to the control cv ‘Hicks’ (Fig 4A). Gene expression analysis (Fig 4B) confirmed high expression of *71cR* in all lines with the exception of Line 9–1 that was used as a low-expression control (Fig 4B).

### Disease resistance and gene silencing in plants transformed with silencing constructs for *CTB1* and *CTB8*

**Fig 5:**
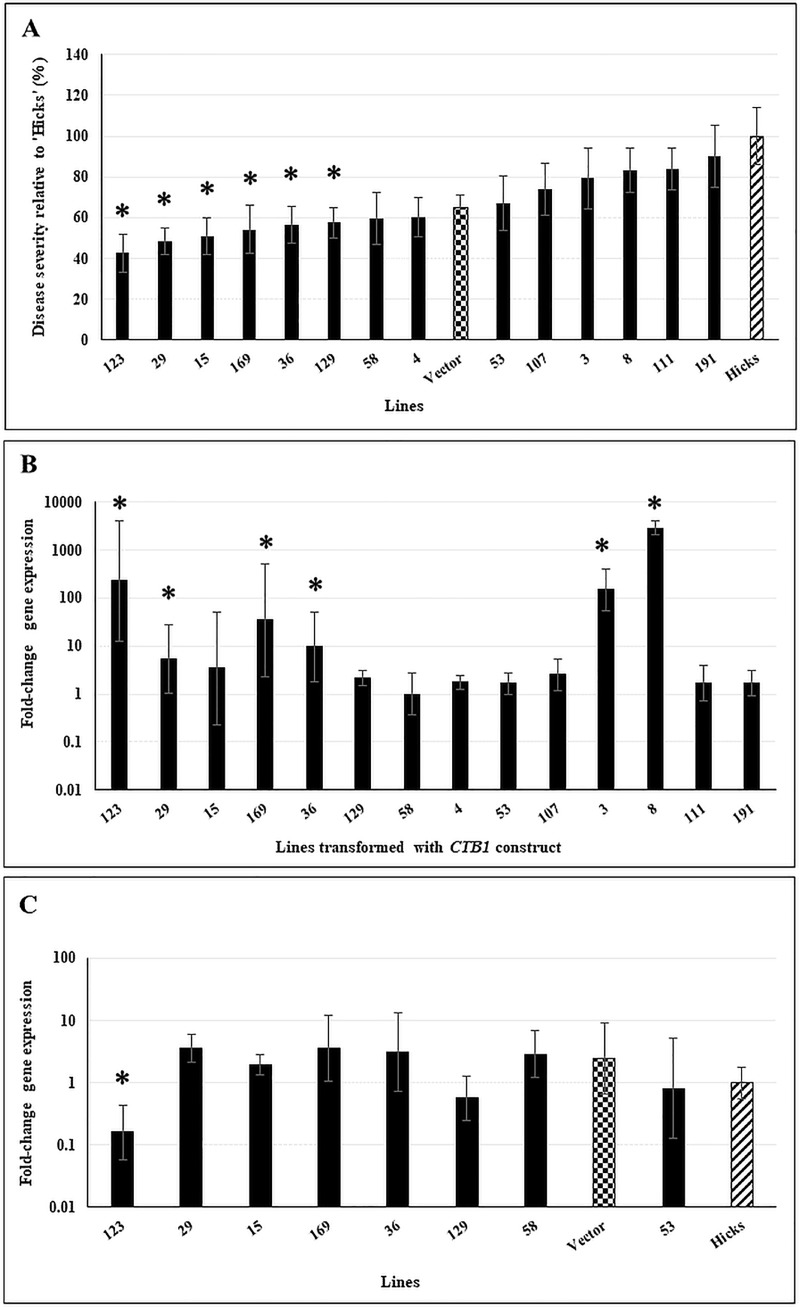
Disease response, transgene expression, and silencing of fungal *CTB1* expression in plants transformed with a silencing construct for *CTB1*. **A.** Disease response of transformed lines inoculated with *C*. *nicotianae*. Controls included a vector-transformed line (checkered bar) and the susceptible tobacco cv ‘Hicks’ (hatched bar). Disease severity was assayed as the number of coalesced lesions at 4 weeks post-inoculation (Fig 2) and are shown relative to severity of cv ‘Hicks’. Data shown are results of two independent experiments with 5 plants/line in each experiment. The within-group differences between the different *CTB1* transformed lines were examined using Satterthwaite-Smith-Welch test. Lines with *p* ≤ 0.01 were considered to be significant (denoted by asterisks). **B.** *CTB1* transgene construct expression in 10-week old non-inoculated plants. Samples were normalized to tobacco polyubiquitin gene expression. Statistical significance was assessed using one-way ANOVA combined with Dunnett’s multiple comparison analysis using the lowest normalized transcript (Line 58) as the control group. Gene expression was considered statistically significant with *p* ≤ 0.05 (denoted by asterisks). Fold-change gene expression is shown on a log_10_ scale relative to the lowest expressor (Line 58). Error bars indicate standard error from three biological replicates. Each biological replicate was tested with three technical replicates. **C.** Expression of fungal *CTB1* in selected infected *CTB1* transformed lines, vector control, and cv ‘Hicks’ at 5-weeks post-inoculation. Controls included a vector-transformed line (checkered bar) and the susceptible tobacco cv ‘Hicks’ (hatched bar). Samples were normalized to fungal actin gene expression. Statistical significance was assessed using one-way ANOVA combined with Dunnett’s multiple comparison analysis using the lowest normalized transcript of ‘Hicks’ as the control group. Gene expression was considered statistically significant with *p* ≤ 0.05 (denoted by asterisks). Fold-change gene expression is shown on a log_10_ scale relative to ‘Hicks’ expression. Error bars indicate standard error from three biological replicates. Each biological replicate was tested with three technical replicates.

Plants transformed with *CTB1* and *CTB8* silencing constructs were inoculated and disease severity was assayed as described above for plants transformed for expression of the cercosporin-resistance genes. Plants transformed to silence *CTB1* showed high levels of resistance compared to cv ‘Hicks’ (Fig 5A), with 6 of 14 lines assayed showing significantly less disease. Symptoms on resistant plants were similar to what was seen with the *ATR1*-transformed plants, with smaller lesions that did not expand (Fig 2). Analysis of transgene expression in non-inoculated plants (Fig 5B) with one-way ANOVA indicated that were significant differences between the normalized transcripts. Post hoc comparisons of normalized transcripts using Dunnett’s test with Line 58 as the control group indicated that the expression of the *CTB1* construct was statistically significant in six lines (*p* ≤ 0.05). Lines 29, 36, 123, and 169 that had statistically significant *CTB1* construct expression were also found to be highly resistant to the pathogen. Although the *CTB1* construct was highly expressed in Lines 3 and 8, no significant disease resistance was found. However, Lines 15 and 129, that did not have statistically significant *CTB1* expression, were found to be highly resistant to the pathogen. Silencing of the fungal *CTB1* was also assayed by examining the fungal *CTB1* transcripts from plants 5-weeks post-inoculation using qPCR (Fig 5C). Results from a one-way ANOVA combined with Dunnett’s test using normalized fungal *CTB1* transcripts from cv ‘Hicks’ as control, revealed that Line 123 had statistically significant lower amount of normalized *CTB1* transcripts (*p* ≤ 0.05). Therefore, Line 123, the most resistant of the lines assayed did show the greatest silencing of *CTB1*; silencing of *CTB1*, however, could not be detected in other lines that showed resistance to disease.

**Fig 6:**
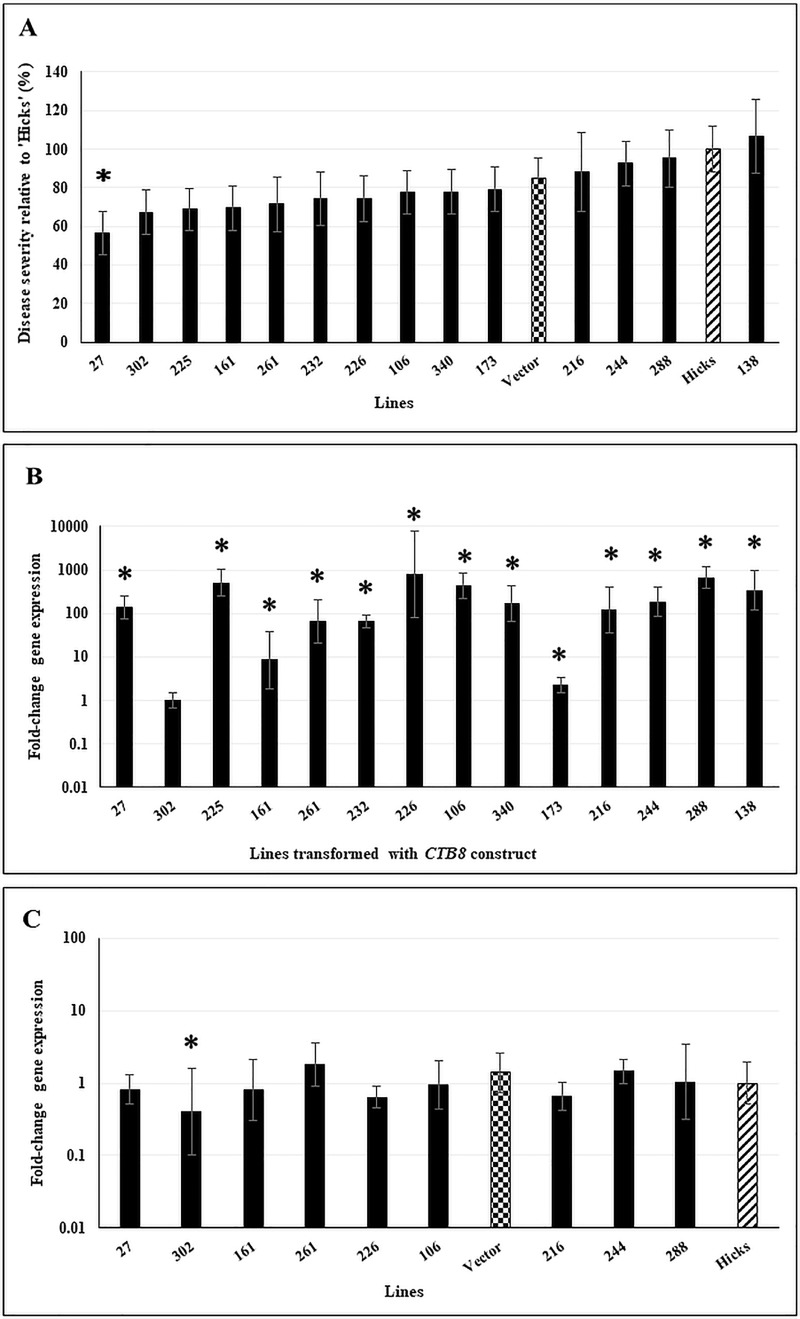
Disease response, transgene expression, and silencing of fungal *CTB1* expression in plants transformed with a silencing construct for *CTB8*. **A.** Disease response of transformed lines inoculated with *C*. *nicotianae*. Controls included a vector-transformed line (checkered bar) and the susceptible tobacco cv ‘Hicks’ (hatched bar). Disease severity was assayed as the number of coalesced lesions at 4 weeks post-inoculation, and are shown relative to severity of cv ‘Hicks’. Data shown are results of two independent experiments with 5 plants/line in each experiment. The within-group differences between the different *CTB8* transformed lines were examined using Satterthwaite-Smith-Welch test. Lines with *p* ≤ 0.01 were considered to be significant (denoted by asterisk). **B.** *CTB8* transgene construct expression in 10-week old non-inoculated plants. Samples were normalized to tobacco alpha-tubulin gene expression. Statistical significance was assessed using one-way ANOVA combined with Dunnett’s multiple comparison analysis using the lowest normalized transcript (Line 302) as the control group. Gene expression was considered statistically significant with *p* ≤ 0.05 (denoted by asterisks). Fold-change gene expression is shown on a log_10_ scale relative to the lowest expressor (Line 302). Error bars indicate standard error from three biological replicates. Each biological replicate was tested with three technical replicates. **C.** Expression of fungal *CTB1* in selected *CTB8-*silenced transformed lines, vector control, and cv ‘Hicks’ at 5-weeks post-inoculation. Controls included a vector-transformed line (checkered bar) and the susceptible tobacco cv ‘Hicks’ (hatched bar). Samples were normalized to fungal actin gene expression. Statistical significance was assessed using one-way ANOVA combined with Dunnett’s multiple comparison analysis using the lowest normalized transcript of ‘Hicks’ as the control group. Gene expression was considered statistically significant with *p* ≤ 0.05 (denoted by asterisks). Fold-change gene expression is shown on a log_10_ scale relative to ‘Hicks’ expression. Error bars indicate standard error from three biological replicates. Each biological replicate was tested with three technical replicates.

Only one of 14 lines assayed that were transformed with the *CTB8* silencing construct showed resistance to disease relative to cv ‘Hicks’ or the vector control (Fig 6A). Transgene expression analysis in non-inoculated plants (Fig 6B) using normalized *CTB8* from Line 302 as a control, revealed that the *CTB8* construct was highly expressed and was statistically significant in all lines (*p* ≤ 0.05). CTB8 is the pathway regulator, and deletion of *CTB8* abolishes expression of all genes in the CTB pathway including *CTB1* [36]. We thus assayed for silencing of *CTB1* as a measure of silencing of the pathway. Results using normalized fungal *CTB1* transcripts from cv ‘Hicks’ as control, revealed that Line 302 had a statistically significant lower amount of normalized *CTB1* transcripts (*p* ≤ 0.05, Fig 6C). Thus, Line 302 that was the lowest expressor of the *CTB8* construct also showed the greatest amount of silencing, although this did not lead to disease resistance.

## Discussion

Extensive studies have documented the importance of the photoactivated toxin cercosporin in disease caused by members of the genus *Cercospora* [12–17]. Given the almost universal toxicity of cercosporin due to its production of singlet oxygen, the strategy of utilizing cercosporin autoresistance genes from the fungus is a promising approach for engineering *Cercospora* resistance in plants [3]. In addition, recent successes with the use of host-induced gene silencing to silence critical fungal pathogenicity factors [35], coupled with the characterization of the cercosporin biosynthetic pathway gene cluster [36], suggest that silencing of cercosporin production may also be a useful strategy. We tested both of these strategies.

In our work to test resistance genes, we selected three genes that were shown in previous studies to be involved in cercosporin autoresistance in *Cercospora*. These genes were identified through a subtractive hybridization strategy between the wild type and a mutant for the CRG1 transcription factor shown to regulate both cercosporin resistance and production [25, 26]. One of the genes recovered through this strategy, *CFP*, was the first cercosporin-resistance gene ever identified. It was initially identified as a light-induced gene involved both in cercosporin production and in resistance [12]. *CFP* has also recently been shown to flank the cercosporin biosynthetic cluster in *C*. *beticola* [37]. *CFP* encodes an MFS transporter, and in previous work we also documented a role for this gene in cercosporin resistance, as over-expression of *CFP* in a cercosporin-sensitive *C*. *nicotianae crg1* mutant increases cercosporin resistance [27]. Previous work had documented reductions in lesion size caused by *C*. *nicotianae* in tobacco cv ‘Xanthi’ transformed with *CFP* [39]. Our *CFP*-transformed lines were not more resistant to *C*. *nicotianae* infection, however, in spite of strong expression of the gene. It is not clear why our results differ from the Upchurch et al. study, but it may be due to genotypic differences in the host species. We used a flue-cured tobacco cv ‘Hicks’, rather than the sun-cured oriental tobacco cultivar ‘Xanthi’. Genome sequence data for these two tobacco types has been published [43].

In contrast to the *CFP* lines, resistance to *C*. *nicotianae* was identified in lines transformed with both *ATR1* and *71cR*. Interestingly, the phenotype of the resistance response differed between the two, with *ATR1*-transformed lines showing a reduction mostly in the size of lesions and the *71cR*-transformed lines showing fewer lesions that did expand, suggesting a difference in the mode of action of these two genes in protecting against infection. The symptoms on the *ATR1-* transformed plants are similar to what is seen when plants are inoculated with cercosporin-deficient mutants. *ATR1* encodes an ABC transporter that plays a role in both cercosporin production and resistance [27]. Cercosporin production in *ATR1* disrupted mutants is approximately 25% of wild-type levels [27]. Mutants are also sensitive to cercosporin, and overexpression of *ATR1* in the aforementioned cercosporin-sensitive *C*. *nicotianae crg1* mutant increases cercosporin resistance [27]. The precise function of ATR1 in cercosporin resistance, however, is not clear. *71cR* encodes a hypothetical protein that is localized in the cytoplasm, has an NTF2-like superfamily DUF1348 domain of unknown function, and has some similarities to the steroid delta-isomerase family of proteins [29]. Expression of *71cR* was induced in a cercosporin-sensitive mutant exposed to cercosporin, suggesting a role in resistance, and subsequent expression of the gene in the cercosporin-sensitive fungus *N*. *crassa* confirmed its ability to impart resistance to cercosporin [29]. However, the precise function of 71cR, either in the fungal life cycle or in providing resistance, is not known. Further work is needed to define the precise role of these genes in imparting resistance.

In addition to expression of cercosporin-resistance genes, we also tested the efficacy of host-induced gene silencing of cercosporin production to provide disease resistance. The CTB gene cluster for cercosporin biosynthesis has been identified and characterized (for review see [4]). The core cluster contains eight genes, encoding the polyketide synthase, a transcription factor, an MFS transporter, as well as for methyltransferases and oxidoreductases. Recent work in *C*. *beticola* identified an additional five genes that flank the CTB cluster [37]. We tested silencing of the CTB1 polyketide synthase [13], the first enzyme in the pathway, and of the CTB8 transcription factor, shown to regulate expression of the cluster genes [36]. Silencing of either gene provided resistance to infection, although greater numbers of resistant lines were obtained with *CTB1* silencing. Symptoms on inoculated *CTB1*-silenced plants, i.e. small lesions that did not expand, resembled symptoms obtained when plants are inoculated with cercosporin-deficient mutants [13, 17], consistent with inhibition of cercosporin production.

Overall, differences in effectiveness between the genes utilized was reflected as the proportion of lines tested that were significantly resistant, rather than the degree of symptom reduction. Lines that showed resistance (those expressing *ATR1* and *71cR* and silenced for *CTB1* and *CTB8*) had 40–60% of the disease severity seen on ‘Hicks’. We did see a difference in the proportion of lines that were resistant, with *71cR-*expressors and *CTB1*-silenced lines having the highest proportion of resistant lines. With the exception of *71cR* line 9–1, which was specifically selected as a control due to its low transgene expression, we did not see a correlation between expression of the transgenes and resistance. This may be due to the fact that we initially selected transformants based on highest expression of the transgene. Lack of correlation between transgene expression and phenotype has previously been reported. For example, Fagoaga and co-workers did not see a correlation between transgene protein expression of the pathogenesis-related protein PR-5 and resistance to *Phytophthora citrophthora* in transgenic orange [44]. Grand et al. found a correlation between the predicted phenotype and expression of preformed defense regulator genes in some but not all transgenic rice lines expressing overexpression constructs, an observation they hypothesized may have been due to expression being above a maximum level needed for the response [45].

In summary, we have tested the efficacy of *Cercospora* cercosporin-resistance genes and of silencing of cercosporin biosynthesis on providing resistance to *Cercospora* disease development. Both strategies were successful in providing resistance. The degree of symptom reduction was similar across strategies, but silencing of *CTB1* and expression of *71cR* provided the highest proportion of lines with statistically significant resistance, and may have utility in engineering crop plants for resistance to this damaging group of pathogens.

## Methods

### Vector construction

An *in vitro* spliced coding sequence of the *ATR1* gene was amplified from the genomic DNA of *C*. *nicotianae* (GenBank accession number EU530631) using the methodology described previously [46]. The PCR amplification using primers with Gateway adapter sequences introduced the *att*B1 and *att*B2 sites at both ends of the 4368-bp spliced *ATR1* amplicon (Table 1). Full length 1821-bp cDNA of the *CFP* gene from *C*. *nicotianae* (GenBank accession number EU530632) was amplified using primers with Gateway adapter sequences (Table 1). The intronless 453-bp *71cR* gene (GenBank accession number KJ126714) was amplified from the genomic DNA of *C*. *nicotianae* with primers carrying the Gateway *att*B adapter sequences (Table 1). After sequencing each of the genes, Gateway BP clonase was used to insert the genes into the donor vector pDONR221, then Gateway LR clonase was used to move the genes into the destination vector pEarleyGate 100 [47] by using the Gateway cloning system according to the manufacturer’s recommendations (ThermoFisher Scientific, Waltham, MA). Constructs were transformed into *E*. *coli* DH5α and then mobilized as previously described [48] into the *Agrobacterium tumefaciens* binary vector strain EHA105 for plant transformation. Transformants were selected based on resistance to kanamycin (Sigma-Aldrich, St. Louis, MO).

**Table 1:** Primers with adaptors used for plasmid construction.

Primer name	Primer sequence ^a^	Accession number
ATR1-ATTB1	**GGGGACAAGTTTGTACAAAAAAGCAGGCTTA**ATGCCTT CCTCCAACGG	EU530631
ATR1-ATTB2	**GGGGACCACTTTGTACAAGAAAGCTGGGTA**CTATTCCG CCTTCTTCGA	
CFP-ATTB1	**GGGGACAAGTTTGTACAAAAAAGCAGGCTTA**ATGGCAA GCCCAGCGCGATC	EU530632
CFP-ATTB2	**GGGGACCACTTTGTACAAGAAAGCTGGGTA**TCACACTGC TTTGCCCGCGATC	
71cR-ATTB1	**GGGGACAAGTTTGTACAAAAAAGCAGGCTTA**CCATCTCA AAATGTCT	KJ126714
71cR-ATTB2	**GGGGACCACTTTGTACAAGAAAGCTGGGTA**CTCACCGGC CTTTGCCTC	
LC1F-CTB1	**CGACGACAAGACCCTT**GAAGAGTGAACTACTTCCAC	AY649543
LC2R-CTB1	**GAGGAGAAGAGCCCT**TATTGAGCAGAACACGACG	
LC1F-CTB8	**CGACGACAAGACCCTT**GGCACTCAATCACCGCCGAT	DQ991510
LC2R-CTB8	**GAGGAGAAGAGCCCT**CAACAATAGCCTGTGGCACCAG	

^a^ Bold and underlined sequences are adaptor sequences

The pRNAi-LIC vector (GenBank accession number GQ870263) was used for cloning *CTB1* and *CTB8* silencing constructs, with expression under the control of the CaMV 35S promoter and kanamycin as the selection marker [49]. To generate *CTB1* ihpRNA construct, the same 798-bp region from the ketosynthase domain of the *C*. *nicotianae CTB1* gene (GenBank accession number AY649543) was amplified twice with two different sets of primers. The first PCR product was obtained using the primers LC1F-CTB1 and LC2R-CTB1 (Table 1). The second PCR product was obtained by using the primers LIC3-TT-LIC2 and LIC4-TT-LIC1 [49] with the purified product of the first PCR serving as the template. Both PCR samples were purified by using the QIAquick PCR purification kit (Qiagen Inc., Germantown, MD). The PCR products along with the vector were mixed and transformed into *E*. *coli* DH5α and then mobilized as previously described [48] into the *A*. *tumefaciens* binary vector strain EHA105 for plant transformation. To generate *CTB8* ihpRNA construct, two sets of PCR products targeting the same 788-bp region of the *C*. *nicotianae CTB8* gene (GenBank accession number DQ991510) were obtained. The first PCR product was obtained using the primers LC1F-CTB8 and LC2R-CTB8 (Table 1). The second PCR product was obtained using the primers LIC3-TT-LIC2 and LIC4-TT-LIC1 with the purified product of the first PCR as the template. The PCR products were purified, transformed into *E*. *coli* DH5α, and then mobilized into the *A*. *tumefaciens* binary vector strain EHA105. Transformants carrying either of the silencing constructs were selected based on resistance to kanamycin.

### Plant transformation

All transformations were done with *N*. *tabacum* cv ‘Hicks’. *ATR1*, *CFP*, *CTB1* and *CTB8*, transformations were done as previously described [24] with *N*. *tabacum* maternal haploids, produced via pollination of *N*. *tabacum* with *N*. *africana* [40]. For *ATR1* and *CFP*, shoots were regenerated on MS medium supplemented with 5mg/l bialaphos (BioWorld, Dublin, OH); for *CTB1* and *CTB8*, shoots were regenerated on the same medium but supplemented with 50 mg/l kanamycin. In all cases, shoots were rooted on MS medium lacking 6-Benzylaminopurine (Sigma-Aldrich, St. Louis, MO) but with bialaphos or kanamycin. Plants were initially screened by PCR to confirm integration of the construct using primers shown in Table 2. Plants were then screened by qPCR to assay gene expression using primers shown in Table 3. Homozygous doubled haploid plants were obtained from selected high-expression transformants via a mid-vein culture technique as previously described [50]. Doubled haploids of each line were selected based on production of pollen and viable selfed-seed, and the T_1_ seed progeny used for all subsequent analyses.

**Table 2:** Primers used for confirming transformation in regenerated plants.

Gene	Primer sequence	Annealing temperature	Amplicon size
*ATR1*	ATGCCTTCCCTCCAACGGTC	58	4290
	CTATTCCGCCTTCTTCGACTGC		
*CFP*	CTCACCCGGGGATGATGGCAAGCCCAGCG	67	1850
	CGCGTCTAGAATTCACACTGCTTTGCCCGCG		
*71cR*	ATGTCTCTCAAGCCACCCTAC	55	230
	CGAATAGTTCTTTGCGAAGGCTGTAG		
*CTB1*	CTGACGTAAGGGATGACGCAC	56	273
	ACGCCATGTGCAATGCTGCC		
*CTB8*	CCACTATCCTTCGCAAGACCC	56	307
	GTTGCAACAGCACTTCCAGCC		

**Table 3:** Primer sequences and reference genes used for RT-qPCR assays. Genes assayed in RT-qPCR assays from Figs 1B, 3B, 4B, 5B, 5C, 6B and 6C are shown in the table along with the forward and reverse primer sequences, two reference genes for each gene of interest, annealing temperature of the primers, and amplicon sizes.

Gene	Primer sequence	Reference gene ^c^	Annealing temperature	Amplicon size
*ATR1*	TGCGAAGCCTGATATGCACCCGTGG	*NtEF1-a*	62	194
	CCTCAGCACCTCTGACACCGGTACAAGC	*NtUbi*		
*CFP*	CAGCGACTGCATTCCAACTA	*NtEF1-a*	52	438
	GCTACAGCTCCGATAGGCAGATTGAT	*NtUbi*		
*71cR*	TCAAGCCACCCTACAATGCCTCAA	*NtEF1-a*	58	160
	TTATTTGGTCGGTGCCTTGGACGA	*NtUbi*		
*CTB1*^a^	CTTACATTTGGATTGATTACAGTTGGTTCC	*NtTubulinA*	54	227
	GCCAAGATCTTGCTTATGCTCAA	*NtUbi*		
*CTB8*	AGAAGATCTGGAGCGCCGTAC	*NtTubulinA*	56	320
	CAAGGGCCCTGAGGAGAAGA	*NtUbi*		
*CnCTB1*^b^	GAGTGGTGGCATTCGGGGATCA	*CnActin*	59	163
	GGCGTCTCTTCTTGTTGCTCGCT	*CnTubulin*		
*NtEF1-a*	CCTGGACACAGGGACTTCATCAAG	N/A^d^	58	159
	GACACCAAGGGTGAAAGCAAGCA			
*NtTubulinA*	GCTGGGAACTTTACTGCCTCGAAC	N/A	58	130
	GGAACATGCTTTCCAGCTCCAGTT			
*NtUbi*	GACATTGACTGGGAAGACCATCACCT	N/A	59	169
	CTGGATATTGTAGTCAGCCAAGGTCCT			
*CnActin*	TGACGATGCGCCACGAGCTGT	N/A	57	376
	TTGATTGGAGCCTCGGTGAGC			
*CnTubulin*	CCCACGTCTCCACTTCTTCATG	N/A	54	103
	CGAAGATTTGCTGGGTGAGC			

^a^ primers for *CTB1* silencing construct expression

^b^ primers for assaying *CTB1* silencing; *CnCTB1* = *C*. *nicotianae CTB1*

^c^ *NtEF1-a* = *N*. *tabacum elongation factor 1-alpha*; *NtUbi* = *N*. *tabacum polyubiquitin*; *NtTubulinA* = *N*. *tabacum alpha tubulin*; *CnActin* = *C*. *nicotianae actin; CnTubulin* = *C*. *nicotianae beta tubulin*

^d^ N/A = not applicable

*71cR* transformation utilized diploid plants of cv ‘Hicks’, and were transformed as described above. Regenerated plants were selected and rooted on medium containing 5mg/l bialaphos. Regenerated T_0_ plants were screened by PCR to confirm transformation, and then by qPCR to quantify *71cR* expression using primers shown in Tables 2 and 3. Selected plants were grown to flowering, allowed to self, and seed was recovered from five transformed plants: four with the highest levels of expression (Lines 4, 7, 14, 20) and one with the lowest gene expression (Line 9) for use as a control. Seed from each plant was screened *in vitro* for bialaphos resistance by plating seeds on MS medium containing 5mg/l bialaphos and incubating at room temperature for 14 days under a 16 hr light/8 hr dark cycle. Ten bialaphos-resistant seedlings (T_1_) from each of the five T_0_ plants were grown in the greenhouse, allowed to self, and T_2_ seed harvested. The T_2_ seed from the 10 plants of each of the five lines were screened *in vitro* for resistance to bialaphos as described above. T_1_ plants producing seed populations that were not segregating for bialaphos resistance (S2 Fig) were scored as homozygous. T_2_ seed from these plants were used for all subsequent analyses.

### Gene expression

Gene expression was used to initially select transformed plants from cultures. Total RNA was isolated from rooted plantlets using TRI Reagent (Sigma-Aldrich, St. Louis, MO). Isolated RNA was treated with Roche’s RNase-free DNase I, (Sigma-Aldrich, St. Louis, MO) to eliminate genomic DNA. Reverse transcription was carried out using 900 ng of total RNA using iScript Reverse Transcription Supermix for RT-qPCR (Bio-Rad, Hercules, CA), according to manufacturer’s recommendations. qPCR was carried out using iQ SYBR Green Supermix (Bio-Rad, Hercules, CA) with a thermal cycling protocol that included an initial denaturation step of 95°C for 2 minutes, followed by 45 cycles of 95°C for 10 seconds, target-dependent annealing temperature for 30 seconds, and 72°C for 30 seconds with a plate read. At the end of each reaction, melt curves were used to verify the amplification of a single, correct product. Each sample reaction was run as triplicates. Normalization was carried out against two tobacco reference genes (Table 3) that had the same efficiency as the target gene, and fold-changes in gene expression was calculated using the 2−^ΔΔCT^ method.

Subsequent gene expression analysis was performed on doubled haploid plants and in the *71cR* transformants selected for homozygosity. Transgenic plants and the wild type cv ‘Hicks’ were grown in the greenhouse in 6-inch pots for 10 weeks. Each transgenic line comprised of three biological replicates. Total RNA was isolated, treated with DNase I, and reverse transcribed as described above. qPCR was conducted as described above. Normalization was carried out against two tobacco reference genes that amplified with the same efficiency as that of the target gene. The heterologous fold-change gene expression was determined by using the 2−^ΔΔCT^ method. The normalized transcript levels of all the samples were expressed relative to the sample with the lowest normalized transcript level. Gene expression was analyzed statistically by one-way ANOVA combined with Dunnett’s multiple comparison post hoc analysis using the lowest expressor as the control group. Statistical analyses were performed using SPSS 26.0 statistical software (IBM, Chicago, IL).

To determine the fate of fungal *CTB1* transcripts in infected plants carrying either the *CTB1* or the *CTB8* silencing constructs, gene expression analysis was evaluated in inoculated transgenic tobacco lines five weeks post inoculation. Total RNA was isolated, treated with DNase I, and reverse transcribed as indicated previously. The cDNA was preamplified using SsoAdvanced PreAmp Supermix (Bio-Rad, Hercules, CA) so as to obtain an unbiased amplification of all target amplicons from the limited amounts of fungal nucleic acid transcripts. The primers for this assay comprised of 50nM of primers that included *CTB1* and fungal reference genes. The preamplification involved a thermal cycling protocol that included an initial denaturation step of 95°C for 3 minutes, followed by 11 cycles of 95°C for 15 seconds and an annealing step of 55°C for 4 minutes. Each preamplified cDNA was diluted with low EDTA TE buffer (USB Corp., Cleveland, OH) and used as the template for qPCR. qPCR was carried out using iQ SYBR Green Supermix with a thermal cycling protocol that included an initial denaturation step of 95°C for 2 minutes, followed by 45 cycles of 95°C for 10 seconds, 55°C for 30 seconds, and 72°C for 30 seconds with a plate read. Primers for detecting the *C*. *nicotianae CTB1* transcripts (*CnCTB1*; Table 3) in the silenced lines were designed to anneal outside the target region for silencing so as to ensure that the transcripts quantified would be of fungal origin rather than those of the T-DNA insertion in the plant containing the silencing region. Normalization was carried out using the fungal actin and tubulin genes, which had the same amplification efficiency as the *CTB1* gene. Statistical significance of normalized fungal transcripts was determined by one-way ANOVA along with Dunnett’s multiple comparison test using ‘Hicks’ as control group. Fold-changes in gene expression was calculated by comparing the fungal transcripts in all transgenic lines including the vector control to that of the wild type ‘Hicks’ by using the 2−^ΔΔCT^ method.

### Disease analysis

Transgenic lines including the vector control and wild type cv ‘Hicks’ were grown in the greenhouse in six inch pots for 12 weeks. Conidia were produced from mycelium of *C*. *nicotianae* isolate ATCC 18366 that was grown on potato dextrose agar for one week, and then homogenized with glass beads, and plated on clarified V8 agar (300 ml of V8 juice supplemented with 4.5g of calcium carbonate, centrifuged for 10 min, the supernatant brought to 1L with water, supplemented with 2% agar) for 7 days at 18°C in the dark. Aerial mycelium was removed by brushing the growth with sterile distilled water so as to expose the stroma. Plates were incubated for an additional week at 18°C in the dark. Conidia were harvested with sterile distilled water supplemented with 0.5% Tween 20 (Sigma-Aldrich Chemicals, St. Louis, MO), and the concentration adjusted to 5 x 10^4^/ml. Spores were atomized onto the abaxial and adaxial sides of tobacco leaves. The entire plant was covered with bags for 4 days to allow symptom development under conditions of high humidity. In each experiment, each transgenic line along with the vector control and wild type cv ‘Hicks’ was represented by five biological replicates, and the entire study was repeated once again. Symptoms were monitored weekly for up to six weeks post inoculation. Disease severity of each line was assessed as previously described [13] as the proportion of the mean number of coalesced lesions on five leaves of each of five plants per line compared to wild type cv ‘Hicks’. To test the role of the transgene in conferring resistance to *C*. *nicotianae*, the disease severity in transgenic lines were compared to the wild type control and the data were statistically analyzed using the Satterthwaite-Smith-Welch test [41, 42].

## Supporting information

S1 Fig Transgene expression in tobacco transformed to express *C*. *nicotianae* cercosporin-resistance genes. **A.** *ATR1* expression in haploid tobacco plants normalized with the tobacco ubiquitin gene. **B.** *CFP* expression in haploid tobacco plants normalized with the tobacco ubiquitin gene. **C.** *71cR* expression in diploid tobacco plants normalized with tobacco elongation factor gene. Expression is shown as fold-change relative to the lowest expressor among the lines.
(TIF)

S2 Fig Screening *71cR* transformed plants for homozygosity via screening seed progeny for resistance to the bialaphos resistance marker. Left: cv ‘Hicks’ showing uniform sensitivity to bialaphos; middle: seed of plant 71cR20-3 showing segregation for bialaphos resistance; right: seed of plant 71cR20-1 showing uniform resistance to bialaphos and scored as homozygous.
(TIF)

## References

[1] Lartey RT, Weiland J, Panella L, Crous P, Windels CE. Cercospora Leaf Spot of Sugar Beet and Related Species. St. Paul, MN: APS Press; 2010.

[2] Daub ME, Ehrenshaft M. The photoactivated *Cercospora* toxin cercosporin: Contributions to plant disease and fundamental biology. Ann Rev Phytopath. 2000;38:461–90. 10.1146/annurev.phyto.38.1.461 11701851

[3] Daub ME, Herrero S, Chung KR. Photoactivated perylenequinone toxins in fungal pathogenesis of plants. FEMS Microbiol Lett. 2005;252:197–206. 10.1016/j.femsle.2005.08.033 16165316

[4] Daub ME, Herrero S, Chung KR. Reactive oxygen species in plant pathogenesis: the role of perylenequinone photosensitizers. Antiox Redox Signaling. 2013;19:970–89. 10.1089/ars.2012.5080 23259634

[5] Daub ME. Cercosporin, a photosensitizing toxin from *Cercospora* species. Phytopathology. 1982;72:370–4.

[6] Daub ME, Hangarter RP. Production of singlet oxygen and superoxide by the fungal toxin, cercosporin. Plant Physiol. 1983;73:855–7. 10.1104/pp.73.3.855 16663313 PMC1066561

[7] Dobrowolski DC, Foote CS. Chemistry of singlet oxygen 46. Quantum yield of cercosporin-sensitized singlet oxygen formation. Angewante Chemie. 1983;95:729–30.

[8] Yamazaki S, Okubo A, Akiyama Y, Fuwa K. Cercosporin, a novel photodynamic pigment isolated from *Cercospora kikuchii*. Agric Biol Chem. 1975;39:287–8.

[9] Daub ME. Peroxidation of tobacco membrane lipids by the photosensitizing toxin, cercosporin. Plant Physiol. 1982;69:1361–4. 10.1104/pp.69.6.1361 16662404 PMC426419

[10] Daub ME, Briggs SP. Changes in tobacco cell membrane composition and structure caused by the fungal toxin, cercosporin. Plant Physiol. 1983;71:763–6. 10.1104/pp.71.4.763 16662903 PMC1066118

[11] Steinkamp MP, Martin SS, Hoefert LL, Ruppel EG. Ultrastructure of lesions produced in leaves of *Beta vulgaris* by cercosporin, a toxin from *Cercospora beticola*. Phytopathol. 1981;71:1272–81.

[12] Callahan T, Rose M, Meade M, Ehrenshaft M, Upchurch R. *CFP*, the putative cercosporin transporter of *Cercospora kikuchii*, is required for wild type cercosporin production, resistance, and virulence on soybean. Mol Plant-Microbe Interact. 1999;12:901–10. 10.1094/MPMI.1999.12.10.901 10517030

[13] Choquer M, Lahey KA, Chen H-L, Cao L, Ueng PP, Daub ME, et al The *CTB1* gene encoding a fungal polyketide synthase is required for cercosporin toxin biosynthesis and fungal virulence in *Cercospora nicotianae*. Molec Plant Microbe Interact. 2005;18:468–76. 15915645 10.1094/MPMI-18-0468

[14] Choquer M, Lee M-H, Bau H-J, Chung K-R. Deletion of a MFS transporter-like gene in *Cercospora nicotianae* reduces cercosporin toxin accumulation and fungal virulence. FEBS Lett. 2007;581:489–94. 10.1016/j.febslet.2007.01.011 17250832

[15] Staerkel C, Boenisch MJ, Kroger C, Bormann J, Schafer W, Stahl D. CbCTB2, and O-methyltransferase is essential for biosynthesis of the phytotoxin cercosporin and infection of sugar beet by *Cercospora beticola*. BMC Plant Biology. 2013;13:50 10.1186/1471-2229-13-50 23517289 PMC3616835

[16] Shim WB, Dunkle LD. *CZK3*, a MAP kinase kinase kinase homolog in *Cercospora zeae-maydis*, regulates cercosporin biosynthesis, fungal development, and pathogenesis. Molec Plant Microbe Interact. 2003;16:760–8. 12971599 10.1094/MPMI.2003.16.9.760

[17] Weiland JJ, Chung KR, Suttle JC. The role of cercosporin in virulence of *Cercospora* spp. to plant hosts. In: Lartey RT, Weiland JJ, Panella L, Crous PW, Windels CE, editors. Cercospora Leaf Spot of Sugar Beet and Related Species. St. Paul, MN: APS Press; 2010 p. 109–17.

[18] Calpouzos L, Stalknecht GF. Symptoms of Cercospora leaf spot of sugar beets influenced by light intensity. Phytopathology. 1967;57:799–800.

[19] Souza AGC, Maffia LA, SIlva FF, Mizubuti ESG, Teixeira H. A time series analysis of brown eye spot progress in conventional and organic coffee production systems. Plant Pathol. 2015;64:157–66.

[20] Bellus D. Physical quenchers of singlet molecular oxygen. Adv Photochem. 1979;11:105–205.

[21] Young AJ. The photoprotective role of carotenoids in higher plants. Physiol Plant. 1991;83:702–8.

[22] Daub ME. Resistance of fungi to the photosensitizing toxin, cercosporin. Phytopathology. 1987;77:1515–20.

[23] Daub ME, Herrero S, Taylor TV. Strategies for the development of resistance to cercosporin, a toxin produced by Cercospora species. In: Lartey RT, Weiland JJ, Panella L, Crous PW, Windels CE, editors. *Cercospora* Leaf Spot of Sugar Beet and Related Species. St. Paul, MN: APS Press; 2010 p. 157–72.

[24] Herrero S, Daub ME. Genetic manipulation of vitamin B-6 biosynthesis in tobacco and fungi uncovers limitations to up-regulation of the pathway. Plant Sci. 2007;172:609–20.

[25] Chung KR, Daub ME, Kuchler K, Schuller C. The *CRG1* gene required for resistance to the singlet oxygen-generating cercosporin toxin in *Cercospora nicotianae* encodes a putative fungal transcription factor. Biochem Biophys Res Commun. 2003;302:302–10. 10.1016/s0006-291x(03)00171-2 12604346

[26] Herrero S, Amnuaykanjanasin A, Daub ME. Identification of genes differentially expressed in the phytopathogenic fungus *Cercospora nicotianae* between cercosporin toxin-resistant and -susceptible strains. FEMS Microbiol Lett. 2007; 275:326–37. 10.1111/j.1574-6968.2007.00903.x 17850326

[27] Amnuaykanjanasin A, Daub ME. The ABC transporter ATR1 is necessary for efflux of the toxin cercosporin in the fungus *Cercospora nicotianae*. Fung Genet Biol. 2009;46:146–58. 10.1016/j.fgb.2008.11.007 19095071

[28] Beseli A, Amnuaykanjanasin A, Herrero S, Thomas E, Daub ME. Membrane transporters in self resistance of *Cercospora nicotianae* to the photoactivated toxin cercosporin. Curr Genet. 2015;61:601–20. 10.1007/s00294-015-0486-x 25862648

[29] Beseli A, Noar R, Daub ME. Characterization of *Cercospora nicotianae* hypothetical proteins in cercosporin resistance. PLOS ONE. 2015; 10.1371/journal.pone.0140676 26474162 PMC4608573

[30] Nowara D, Gay A, Lacomme C, Shaw J, Ridout C, Douchkov D, et al HIGS: Host-induced gene silencing in the obligate biotrophic fungal pathogen *Blumeria graminis*. Plant Cell. 2010;22:3130–41. 10.1105/tpc.110.077040 20884801 PMC2965548

[31] Qi T, Zhu X, Tan C, Liu P, Guo J, Kang Z, et al Host-induced gene silencing of an important pathogenicity factor *PsCPK1* in *Puccinia striiformis* f. sp. *tritici* enhances resistance of wheat to stripe rust. Plant Biotechnol J. 2018;16:797–807. 10.1111/pbi.12829 28881438 PMC5814584

[32] Koch A, Kumar N, Weber L, Keller H, Imani J, Kogel KH. Host-induced gene silencing of cytochrome P450 lanosterol C14 alpha-demethylase-encoding genes confers strong resistance to *Fusarium* species. Proc Natl Acad Sci. 2013;110:19324–9. 10.1073/pnas.1306373110 24218613 PMC3845197

[33] Zhang T, Jin Y, Zhao JH, Gao F, Zhou BJ, Fang YY, et al Host-induced gene silencing of the target gene in fungal cells confers effective resistance to the cotton wilt disease pathogen *Verticillium dahliae*. Molec Plant 2016;9:939–42. 26925819 10.1016/j.molp.2016.02.008

[34] Andrade CM, Tinoco MLP, Rieth AF, Maia FCO, Aragao FJL. Host-induced gene silencing in the necrotrophic fungal pathogen *Sclerotinia sclerotiorum*. Plant Pathol. 2016;65:626–32.

[35] Ghag SB. Host induced gene silencing, an emerging science to engineer crop resistance against harmful plant pathogens. Physiol Mol Plant Pathol. 2017;100:242–54.

[36] Chen H, Lee M-H, Daub ME, Chung K-R. Molecular analysis of the cercosporin biosynthetic gene cluster in *Cercospora nicotianae*. Mol Microbiol. 2007;64:755–70. 10.1111/j.1365-2958.2007.05689.x 17462021

[37] de Jonge R, Ebert MK, Huitt-Roehl CR, Pal P, Suttle JC, Spanner RE, et al Gene cluster conservation provides insight into cercosporin biosynthesis and extends production to the genus *Colletotrichum*. Proc Natl Acad Sci. 2018; 115:E5459–E5466. 10.1073/pnas.1712798115 29844193 PMC6004482

[38] Newman AG, Townsend CA. Molecular characterization of the cercosporin biosynthetic pathway in the fungal plant pathogen *Cercospora nicotianae*. J Am Chem Soc. 2016;138:4219–28. 10.1021/jacs.6b00633 26938470 PMC5129747

[39] Upchurch RG, Rose MS, Eweida M, Zuo W. Expression of the cercosporin transporter, *CFP*, in tobacco reduces frog-eye lesion size. Biotechnol Lett. 2005;27:1543–50. 10.1007/s10529-005-1780-3 16245172

[40] Burk LG, Gerstel DU, Wernsman EA. Maternal haploids of *Nicotiana tabacum* L. from seed. Science. 1979;206:585 10.1126/science.206.4418.585 17759429

[41] Derrick B, Toher D, White P. Why Welch’s test is Type I error robust. The Quantitative Methods in Psychology. 2016;12:30–8.

[42] Welch BL. The generalization of “student’s” problem when several different population variances are involved. Biometrika. 1947;34:28–35. 10.1093/biomet/34.1-2.28 20287819

[43] Sierro N, Battey JND, Ouadi S, Bakaher N, Bovet L, Willig A, et al The tobacco genome sequence and its comparison with those of tomato and potato. Nature Communications. 2014;5:3833 10.1038/ncomms4833 24807620 PMC4024737

[44] Fagoaga C, Rodrigo I, Conejero V, Hinarejos C, Tuset JJ, Arnau J, et al Increased tolerance to *Phytophthora citrophthora* in transgenic orange plants constitutively expressing a tomato pathogenesis related protein PR-5. Molec Breeding. 2001;7:175–85.

[45] Grand X, Espinoza R, Michel C, Cros S, Chalvon V, Jacobs J, et al Identification of positive and negative regulators of disease resistance to rice blast fungus using constitutive gene expression patterns. Plant Biotechnol J. 2012;10:840–50. 10.1111/j.1467-7652.2012.00703.x 22607456

[46] Davies WL, Carvalho LS, Hunt DM. SPLICE: A technique for generating in vitro spliced coding sequences from genomic DNA. BioTechniques. 2007;43:785–9. 10.2144/000112588 18251255

[47] Earley K, Haag JR, Pontes O, Opper K, Juehne T, Song K, et al Gateway-compatible vectors for plant functional genomics and proteomics. Plant J. 2006;45:616–29. 10.1111/j.1365-313X.2005.02617.x 16441352

[48] Daub ME, Jenns AE, Urban LA, Brintle SC. Transformation frequency and foreign gene expression in burley and flue-cured cultivars of tobacco. Tob Sci. 1994;38:51–4.

[49] Xu G, Sui N, Tang Y, Xie K, Lai Y, Liu Y. One-step, zero-background ligation-independent cloning intron-containing hairpin RNA constructs for RNAi in plants. New Phytol. 2010;187:240–50. 10.1111/j.1469-8137.2010.03253.x 20406406

[50] Kasperbauer MJ, Collins GH. Reconstitution of diploids from leaf tissue of anther-derived haploids in tobacco. Crop Sci. 1972;12:98–101.

