# Supplementary figures and images for: Engineering Cercospora disease resistance via expression of Cercospora nicotianae cercosporin-resistance genes and silencing of cercosporin production in tobacco
Source: PLoS One. 2020 Mar 16;15(3):e0230362. doi: 10.1371/journal.pone.0230362 (PMC7075572; doi:10.1371/journal.pone.0230362)

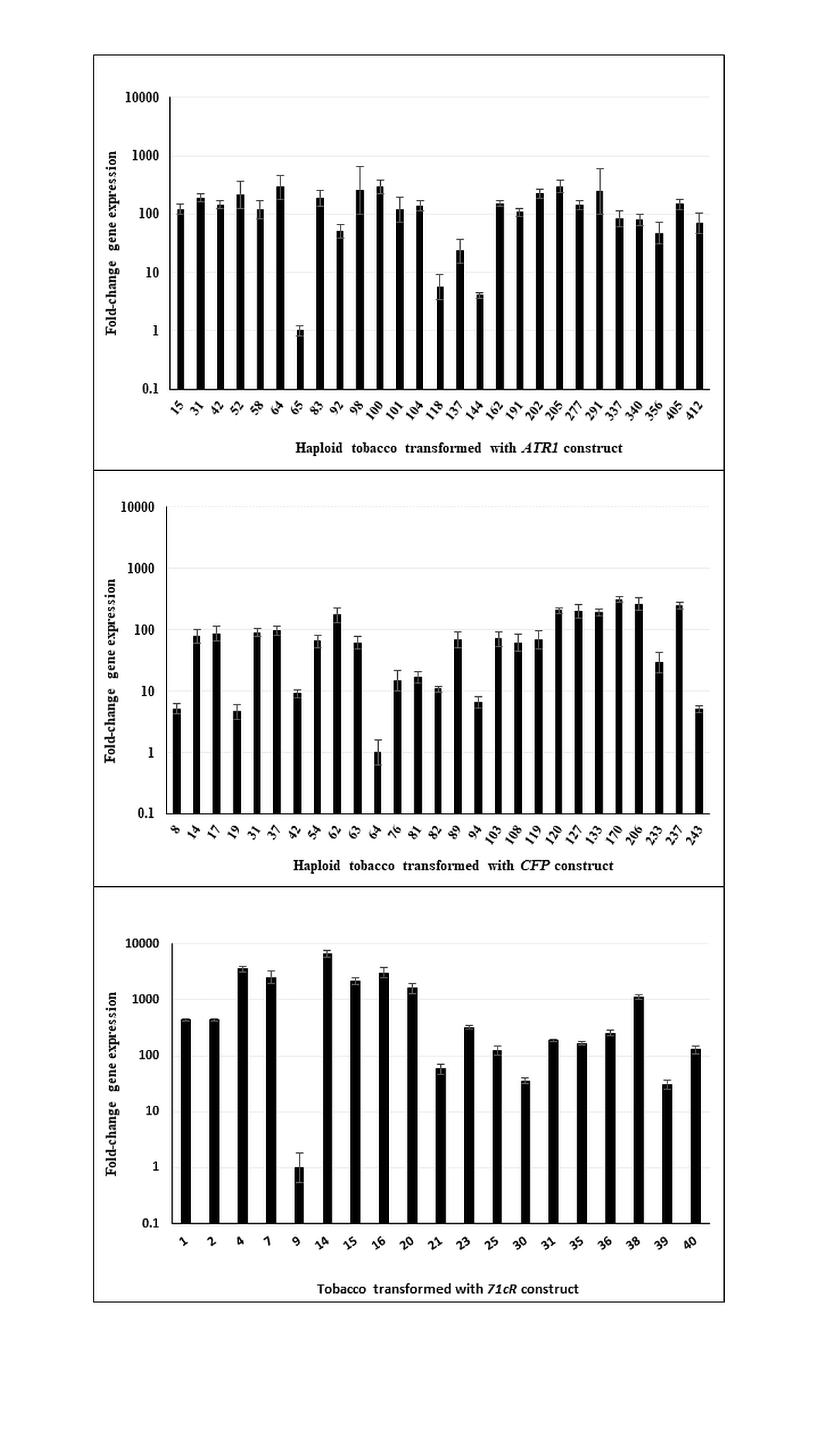

Supplement: S1 Fig — A. ATR1 expression in haploid tobacco plants normalized with the tobacco ubiquitin gene. B. CFP expression in haploid tobacco plants normalized with the tobacco ubiquitin gene. C. 71cR expression in diploid tobacco plants normalized with tobacco elongation factor gene. Expression is shown as fold-change relative to the lowest expressor among the lines. (TIF) [file pone.0230362.s001.tif]

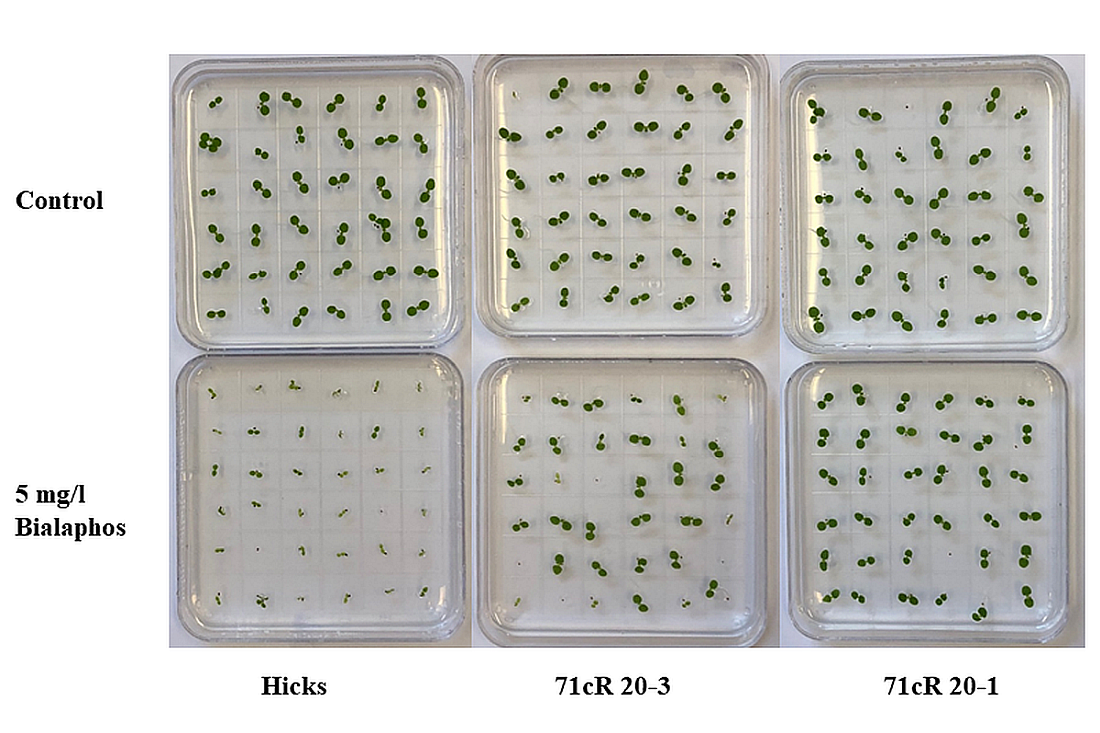

Supplement: S2 Fig — Left: cv ‘Hicks’ showing uniform sensitivity to bialaphos; middle: seed of plant 71cR20-3 showing segregation for bialaphos resistance; right: seed of plant 71cR20-1 showing uniform resistance to bialaphos and scored as homozygous. (TIF) [file pone.0230362.s002.tif]
